# Pock forming ability of fowl pox virus isolated from layer chicken and its adaptation in chicken embryo fibroblast cell culture

**DOI:** 10.14202/vetworld.2015.245-250

**Published:** 2015-03-04

**Authors:** Varsha Rani Gilhare, S. D. Hirpurkar, Ashish Kumar, Surendra Kumar Naik, Tarini Sahu

**Affiliations:** 1 Department of Veterinary Microbiology, College of Veterinary Science and Animal Husbandry, Anjora Durg, Chhattisgarh, India; 2 Department of Animal Nutrition, College of Veterinary Science and Animal Husbandry, Anjora Durg, Chhattisgarh, India

**Keywords:** chicken embryo fibroblast cell culture, chorioallantoic membrane, embryonated egg, fowl pox virus

## Abstract

**Aim:**

The objective of the present study was to examine pock forming ability of field strain and vaccine strain of fowl pox virus (FPV) in chorioallantoic membrane (CAM) of embryonated chicken eggs and its adaptation in chicken embryo fibroblast (CEF) cell culture.

**Materials and Methods:**

Dry scabs were collected from 25 affected birds in glycerin-saline and preserved at 4°C until processed. Virus was isolated in 10-day-old embryonated chicken eggs by dropped CAM method. The identity of the virus is confirmed by clinical findings of affected birds, pock morphology and histopathology of infected CAM. In addition one field isolate and vaccine strain of FPV was adapted to CEF cell culture. CEF cell culture was prepared from 9-day-old embryonated chicken eggs.

**Result:**

Clinical symptoms observed in affected birds include pox lesion on comb, wattle, eyelids and legs, no internal lesions were observed. All field isolates produced similar findings in CAM. Pocks produced by field isolates ranged from 3 mm to 5 mm at the third passage while initial passages edematous thickening and necrosis of CAM was observed. Pocks formed by lyophilized strain were ranges from 0.5 mm to 2.5 mm in diameter scattered all over the membrane at the first passage. Intra-cytoplasmic inclusion bodies are found on histopathology of CAM. At third passage level, the CEF inoculated with FPV showed characteristic cytopathic effect (CPE) included aggregation of cells, syncytia and plaque formation.

**Conclusion:**

FPV field isolates and vaccine strain produced distinct pock lesions on CAMs. Infected CAM showed intracytoplasmic inclusion bodies. The CEF inoculated with FPV field isolate as well as a vaccine strain showed characteristic CPE at third passage level.

## Introduction

Fowl pox is a contagious disease of domestic and wild birds [1] of all ages, sexes and breeds [2] which is caused by fowl pox virus (FPV), a DNA virus that comes under the genus *Avipoxvirus* of family *Poxviridae* and subfamily *Chordopoxvirinae* [3]. FPV is brick shaped [4], has large size genome approximately 288-300 k base pairs (Kbp) [5]. Replication and maturation of the virus occur in the cytoplasm of host cell [6]. Virus are spread by insects [7] and wild birds [8]. Clinically the affected birds show three forms of the disease namely; the cutaneous, diphtheritic and systemic form [9,10]. In the cutaneous form, bird shows nodular lesion on unfeathered parts of the body [1]. The characteristics feature of a diphtheritic form is fibro-necrotic lesions in the mucous lining of the oropharyngeal route [2] and the internal tissues are found to be most affected in the third form [9].

The great concern is needed as the disease causes heavy economic loss. The mortality rate increased up to 50% [11] when the diphtheritic form is accompanied by secondary bacterial infection [12]. For the appropriate diagnosis, viruses are isolated either in cell culture, or embryonated chicken eggs using CAM route or by the combination of both techniques [13,14]. Fowl pox is an emerging disease [15] and the variant FPV has been reported broadly [16]. Disease treatments for fowl pox are not available.

In the present study occurrence of fowl pox was recorded in Durg district of Chhattisgarh. Samples were collected, and the virus was isolated in chorioallantoic membrane (CAM) of embryonated chicken egg and identified on the basis of clinical findings, pock morphology, histopathology of infected CAM. Also one field sample and one commercial vaccine strain were adopted in chicken embryo fibroblast cell culture.

## Materials and Methods

### Ethical approval

The present study was approved by Institutional Animal Ethics Committee.

### Viruses

Between November 2011 and April 2012, fowl pox outbreaks occurred in Durg district of Chhattisgarh. Clinically all affected birds showed crust or nodular lesion on comb, wattles, eyelid, legs and another unfeathered body part. Scab (nodular lesions) was collected aseptically from 25 layer chickens have clinical symptoms. All samples were kept in screw tied vials and stored at 4°C. Laboratory work was conducted at the department of Veterinary Microbiology, Anjora, Durg, Chhattisgarh. Commercially available FPV vaccine strain obtained from Venkateshwara Hatcheries Pvt. Ltd, Malkhed, Tal-Haveli, Pune was also used as a source of vaccine virus and stored at 4°C until required. It was processed simultaneously along with the field strain.

### Sample preparation and virus isolation

Scab collected from afflicted birds were grounded separately in sterilized mortar and pestle and suspended in phosphate buffered saline (PBS) to make ten per cent suspension. The suspension was clarified by centrifugation at 1500 rpm for 15 min and supernatant was treated with a mixture of Penicillin (10000 units per ml of supernatant) and streptomycin (10000 µg per ml of supernatant) for 45 min at 37°C. One vial of freeze-dried vaccine strain (1000 doses) was reconstituted in 1.5 ml PBS. All the field and vaccine (0.2 ml) sample were initially inoculated onto the CAM of specific-pathogen-free 10-day-old developing chicken embryos as described by Cunningham [17]. Following inoculation, the embryos were incubated at 37°C and checked daily for mortality. 5 days of post inoculation, (PI) CAM were harvested and examined for pock lesion. Subsequent passages were required for adaptation of the virus in CAM.

### Histopathology of infected CAM

To verify the identity of the pathogen, pocks on the CAMs were subjected to histological examination. CAM s showing lesions were fixed in 10% buffered formalin solution. The fixed CAMs were embedded in paraffin and cut 4-6 µm thick. Sections were stained with hematoxylin and eosin and examined microscopically for the presence of cytoplasmic inclusion bodies.

### Chicken embryo fibroblast cell culture

Blind passaging of one sample of field strain and vaccine strain of fowl pox was performed in chicken embryo fibroblast cell culture. The primary fibroblast cell culture was prepared from 9-day-old chicken embryos according to the method described by Cunningham [17], using minimum essential medium (Eagle). Uniform suspensions of cells were obtained by the trypsin digest method described by Younger [18]. The uniform cell sheets were formed after 36-48 h of incubation

### Adaptation of FPV in CEF cell culture

Both virus samples were propagated in the CEF cell culture. The monolayers in Leighton’s tubes (5 culture tubes) were inoculated with 0.2 ml of the viruses (10% suspension of CAM). After 2 h of virus adsorption at 37°C the excess inoculum from tubes removed and maintenance medium was then added and incubated at 37°C. After 5 days of PI the medium from five inoculated tubes were pooled after three cycles of alternate freezing and thawing. A portion of the pooled sample was inoculated into a fresh batch of 5 five cell culture tubes at 0.2 ml each tube while a representative portion was preserved at −20°C.

## Results

### Morphology of CAM

**Figure-1:**
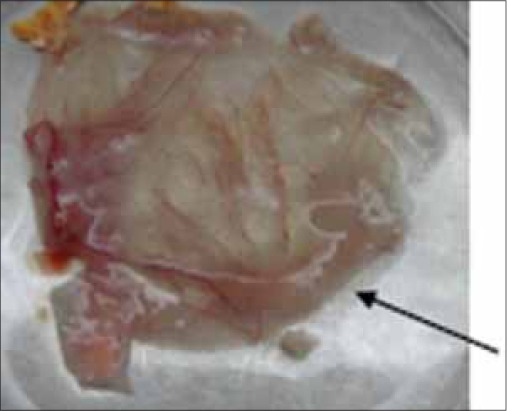
Oedematous thickening of chorioallantoic membrane produced by field at second passage level.

**Figure-2:**
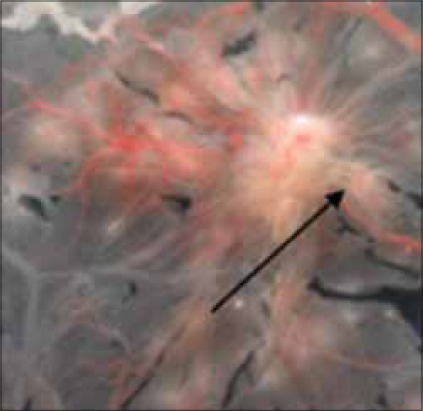
Pock produced by field strain of fowl pox virus (FPV) at third strain of FPV at third passage level.

**Figure-3:**
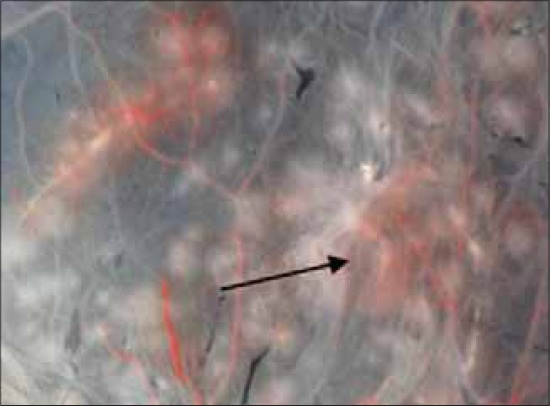
More distinct pocks were produced by field strain of fowl pox virus on chorioallantoic membrane at fourth passage level.

**Figure-4:**
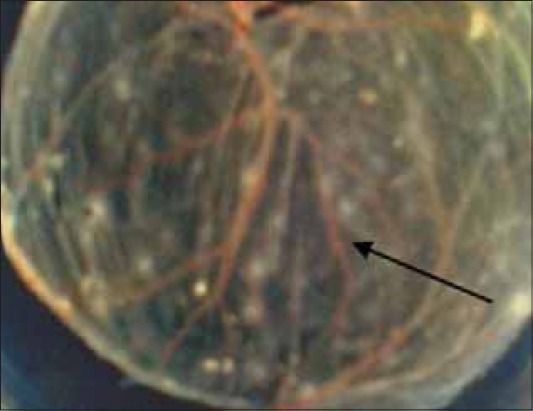
Pocks produced by vaccine strain of fowl pox virus at first passage.

**Figure-5:**
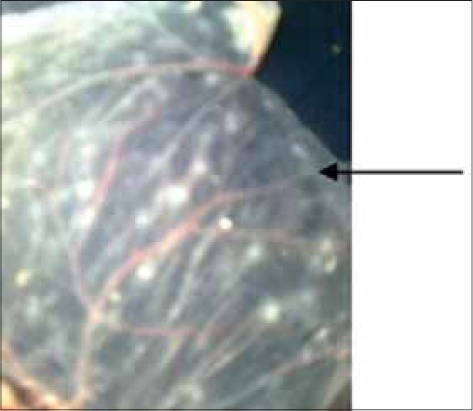
Pocks produced by vaccine strain of fowl pox virus at third passage.

At the gross examination characteristics, pock lesions were observed in fowl pox infected CAMs. At initial passage, the CAM becomes opaque, edematous and thick (Figure-1). Round shape opaque raised necrosed area of 3-5 mm in diameter were developed at the third passage (Figures-2 and 3). Infected CAMs also showed congestion and sometimes small hemorrhagic areas. The pocks that formed by vaccine strain were grayish white and slightly raised sizes ranges from 0.5 mm to 2.5 mm in diameter (Figures-4 and 5). Central area is slightly raised than periphery in pocks. However, mortality was not seen in embryo during the study.

### Histopathology of CAM

**Figure-6:**
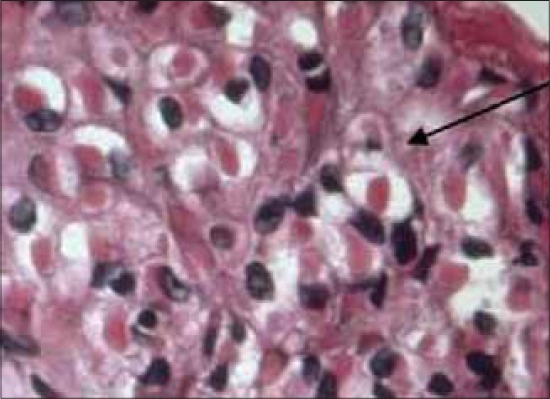
Chorioallantoic membrane showing intra-cytoplasmic inclusion bodies (×100).

Similar findings were observed in all infected CAMs on histopathology. Hyperplasia and hypertrophy of epithelium cells occurred. CAM s becomes two are three folds thicker than the normal CAM. Intra-cytoplasmic inclusions of variable size are found on histopathology of infected CAM (Figure-6).

### Chicken embryo fibroblast cell culture

**Figure-7:**
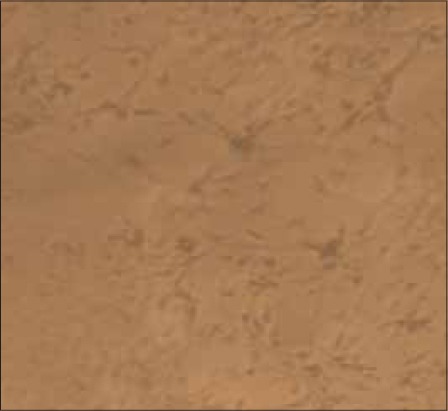
Cytopathic effect produced by vaccine strain of fowl pox virus after 96 h of PI (×10).

**Figure-8:**
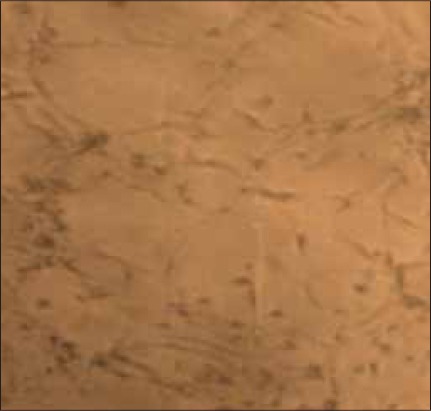
Cytopathic effect produced by field strain of fowl pox virus after 96 h of PI (×10)

The CEF inoculated with FPV showed no characteristic CPE up to the second passage level. At third passage level CEF cell culture showed aggregation of cells which progressed rapidly and appeared as floating cells at 72 h PI. The plaques were formed within 96 h PI. There were rounding and degeneration of cells and appeared as “bunch of grapes.” Massive detachments of cells were observed at 120 h PI. The remaining cells become elongated whereas corresponding uninfected controls showed no such changes. In CEF cultures, vaccine strain produced plaques with a clear centre and less clear peripheral zone (Figure-7). Larger plaques were produced by field strain (Figure-8). On subsequent passage, the CPE was observed earlier. During this study, it was observed that both field strain, as well as vaccine strain of FPV, was propagated successfully in CEF culture.

## Discussion

Fowl pox outbreaks are reported throughout the world, but the incidences vary according to geographical areas [11]. In the present study scabs, samples were taken from 25 affected layer chickens. Cutaneous form was observed in all birds. There was no case of diphtheritic and systemic forms. Variable sizes nodular lesion was observed in skin of unfeathered body part of sick birds, and also they revealed dull, depressed, anorexia, weight loss, decreased egg production and impaired fertility. Current findings are corroborated by others [19,20]. Biswas *et al*. [16] observed FPV infection in unvaccinated backyard chickens without mortality and also pin head size raised lesions were observed in the mucosal surface of the pharynx.

In the present study, fields strain as well as a vaccine strain was inoculated in CAM. All the samples produced characteristic pock lesions on CAM of 10-day-old embryos. In initial passage, the field isolates of FPV produced edema, thickening and necrosis of CAM while characteristics pock lesions appeared from a third passage onwards. Pocks were large opaque white necrosed area on CAM and size ranges from 3 mm to 5 mm in diameter. The centre area of pocks lesion was slightly raised rather than the periphery. The lesions produced by field isolates were diffuse and distributed over the entire CAM. The pocks that formed by lyophilized strain were ranges from 0.5 mm to 2.5 mm in diameter scattered all over the membrane at the first passage, more distinct lesion was obtained in further passages. The virulence of the virus increased with each passage and showed distinct lesions. Result of the present study clearly indicated that pock lesions of variable diameter were appeared on the CAM of scab sample inoculated embryonated chicken eggs. More severe changes were observed in CAM after passaging, indicated adaptation of FPV *via* CAM routes. However, mortality was not seen in embryo during the study. Our findings with regards to the field strain were in agreement with those reported by [11,21]. Our findings with respect to the vaccine strain were in accordance with [22,23]. Although the development of pock lesion on CAM in FPV infected chicken embryo is highly consistent feature, it is always unpredictable about the number of passages required for adaptation [24]. In the present study, local FPV field strain produced distinct pock lesion of constant diameter and, therefore, can be used for neutralization test for evaluating vaccination trials. Specific pathogen free eggs as employed by Prukner-Radovcic *et al*. [25] may not be required.

In the present study, intracytoplasmic inclusions of variable size are found on histopathology of infected CAMs. Similar findings were also reported by Yoshikawa and Alam [26]. Histologically, proliferative and necrotic dermatitis with eosinophilic ring-shaped cytoplasmic inclusions (Bollinger bodies) and clumps of gram-positive cocci (*Staphylococcus hyicus*) were noted in the affected birds [27] and eosinophilic inclusions (Bollinger bodies) on histologic examinations in the cytoplasm of keratinocytes were perceptible [15]. At the histopathological examination, intra-cytoplasmic inclusion bodies and ballooning degeneration were detected in the hyperplastic and hypertrophic epithelial cells [21,28].

Studies were conducted on the pathogenicity of these viruses in the host systems; the virus produced characteristic CPE in CEF cell culture. The CEF cell line inoculated with FPVs showed no characteristic CPE up to secondpassage level. At third passage level CEF, cell culture showed aggregation of cells and floating cells at 72 h PI. After 96 h PI rounding, degeneration of cell was more marked in the form of “bunch of grapes” and 120 h PI, massive detachment of cells was observed. Yadav *et al*. [28] also reported that FPV could be adapted and propagated in CEF cell culture, which produced characteristic CPE (plaque/syncytia formation). It is well documented in previous literature that adaptation of the virus to the appropriate host system restores fully immunogenic potential without losing many antigens [29]. Hence, the approach of prospective development of FPV vaccine, as suggested by Dabas et al. [30], appear to be modest. Through such efforts the problem of vaccine failure, a breakdown of immunity, non-availability of vaccine, cost, etc. can be partially solved.

## Conclusion

All FPV samples produced pock lesion on CAM. At third passage, FPV field isolates produced distinct pock lesions measuring 3-5 mm in diameter whereas smaller sizes (0.5-2.5 mm) pocks were produced by vaccine strain at the first passage. On histopathological examination of all infected CAMs showed variable sizes intra-cytoplasmic inclusion bodies. The CEF inoculated with FPV showed characteristic CPE at third passage level. In CEF cultures, vaccine strain produced plaques with a clear centre and less clear peripheral zone. Larger plaques were produced by field strain than vaccine strain.

## Authors’ Contributions

SDH designed the experiment. VRG carried out the experiment and drafted the final manuscript. AK, SKN and TS helped in the sample collection and analysis. All authors read and approved the final manuscript.
